# Histone Methyltransferase EZH2: A Potential Therapeutic Target for Kidney Diseases

**DOI:** 10.3389/fphys.2021.640700

**Published:** 2021-02-18

**Authors:** Tingting Li, Chao Yu, Shougang Zhuang

**Affiliations:** ^1^Department of Nephrology, Shanghai East Hospital, Tongji University School of Medicine, Shanghai, China; ^2^Department of Medicine, Alpert Medical School and Rhode Island Hospital, Brown University, Providence, RI, United States

**Keywords:** acute kidney injury, chronic kidney disease, diabetic nephropathy, renal cell carcinoma, epigenetic regulation, histone methyltransferase (HMT), enhancer of zeste homolog 2 (EZH2), renal fibrosis

## Abstract

Enhancer of zeste homolog 2 (EZH2) is a histone-lysine N-methyltransferase enzyme that catalyzes the addition of methyl groups to histone H3 at lysine 27, leading to gene silencing. Mutation or over-expression of EZH2 has been linked to many cancers including renal carcinoma. Recent studies have shown that EZH2 expression and activity are also increased in several animal models of kidney injury, such as acute kidney injury (AKI), renal fibrosis, diabetic nephropathy, lupus nephritis (LN), and renal transplantation rejection. The pharmacological and/or genetic inhibition of EZH2 can alleviate AKI, renal fibrosis, and LN, but potentiate podocyte injury in animal models, suggesting that the functional role of EZH2 varies with renal cell type and disease model. In this article, we summarize the role of EZH2 in the pathology of renal injury and relevant mechanisms and highlight EZH2 as a potential therapeutic target for kidney diseases.

## Introduction

Epigenetics refers to the heritable change of gene function and phenotype without alteration in a genes DNA sequence (Berger et al., 2009). The core function of epigenetics is to control access to the DNA genetic code both spatially and temporally to ensure orderly gene expression and silencing according to external signals. Epigenetic regulation involves DNA methylation, histone modification, chromatin recombination, and non-coding RNA (Rosner and Hengstschlager, 2012; Roy and Majumdar, 2012). These modifications control the fate of cells, regulate the normal growth, and development of individuals, and are closely related to the occurrence and development of various diseases such as cancers, diabetes, heart disease, and intestinal disease (Filion et al., 2010). The importance and diversity of histone post-translational modification have been widely studied in many epigenetic regulatory mechanisms. Histone modifications include acetylation, methylation, ubiquitination, hydroxylation, phosphorylation, and ADP ribosylation (Graff and Tsai, 2013; Hyun et al., 2017; Bochyńska et al., 2018; Worden et al., 2019). Methylation can occur in histone and non-histone proteins. Histones are proteins that are abundant in lysine and arginine and are found in eukaryotic cell nuclei. Among four core histone proteins (H2A, H2B, H3, and H4), histone H3 is one of the most important epigenetic markers in transcriptional regulation. The methylation of histone H3 mainly occurs on the arginine (R) and lysine (K) residues in its tail (Wei et al., 2009). Histone arginine residues are monomethylated or dimethylated (Blanc and Richard, 2017), while histone lysine can be monomethylated (me1), dimethylated (me2), or trimethylated (me3). Different methylation sites such as H3K4, H3K9, H3K27, H3K36, and H3K79 have been identified (Wang et al., 2009). Among those sites, trimethylation of lysine 27 in histone H3 (H3K27me3) is a key marker of gene silencing, found mainly in gene promoter and enhancer regions (Duan et al., 2020).

H3K27 trimethylation is mainly carried out by Polycomb Repressor Complex (PRC2; Tie et al., 1998; Sanchez-Beato et al., 2006; Yu et al., 2007; Veneti et al., 2017). PRC2 is a histone methyltransferase that mainly trimethylates the histone H3 lysine 27 (H3K27me3; Margueron and Reinberg, 2011; Di Croce and Helin, 2013). The PRC2 complex consists of four components: enhancer of zeste homolog 1 (EZH1) or enhancer of zeste homolog 2 (EZH2), compressor of zeste12 (Suz12), embryonic ectoderm development (EED), and RbAp46/48. EZH1 and EZH2 are the core components of PRC2, while EED can interact with EZH1 or EZH2 to maintain enzyme activity (Kim et al., 2013; Eich et al., 2020). EZH2 is an essential component of PRC2 methylation activity; it can also methylate some non-histone substrates, such as actin, GATA binding protein 4 (GATA4), android receiver (AR), and estrogen receiver (ER). EZH2 can silence tumor suppressor genes and play an important role in cell aging, fate selection, and differentiation (Jacobs and van Lohuizen, 2002; Plath et al., 2003; Martinez and Cavalli, 2006; Aloia et al., 2013). In addition, EZH2-mediated methylation of non-histones such as STAT3 is an important post-translational modification involved in many life processes, such as the cell cycle, DNA repair, cell aging, differentiation, apoptosis, and tumorigenesis (Kim et al., 2013; Eich et al., 2020).

Enhancer of zeste homolog 2 was discovered in 1996 using yeast two-hybrid experiments (Hobert et al., 1996) and its gene is located on the human chromosome 7q35. EZH2 occupies nearly 40 kb in the gene structure, which contains 20 exons, and the open reading frame is distributed on 19 exons (Cardoso et al., 2000). Studies have shown that EZH2 can inhibit the expression of tumor suppressor genes in normal cells, thereby promoting the abnormal proliferation of cells, and stimulating the metastasis of tumor cells. EZH2 exhibits gene amplification and higher levels of expression in many human malignancies, such as gastric cancer, colon cancer, breast cancer, lymphoid hematopoietic tumors, liver cancer, and nephroblastoma (Su et al., 2003; Mimori et al., 2005; Raman et al., 2005; Sudo et al., 2005; Matsukawa et al., 2006; Saramaki et al., 2006; Shi et al., 2007), and is closely related to tumorigenesis and tumor progression.

Increasing evidence shows that EZH2 is associated with a variety of kidney diseases and pathology. In addition to the abnormal expression and activation of EZH2 in renal tumors, its expression levels and activity were also increased in acute kidney injury (AKI; Zhou et al., 2018b), renal fibrosis (Zhou et al., 2016), diabetic nephropathy (DN; Jia et al., 2019b), lupus nephritis (LN; Rohraff et al., 2019), hyperuricemic nephropathy (Shi et al., 2019), and transplanted and aging kidneys (Li et al., 2016; Han and Sun, 2020). Moreover, pharmacological or genetic inhibition of EZH2 can interfere with pathologic fibrosis in these animal models of kidney disease. This article reviews the role of EZH2 in the pathology of renal disease and relevant mechanisms. We also highlight EZH2 as a potential target for ameliorating fibrosis in kidney disease (Table 1).

**TABLE 1:** EZH2 inhibition on kidney diseases in various in vitro and in vivo models.

**Inhibitors or Knockout mice**	**Models**	**Effects and mechanisms**	**References**
shEZH2, 3-DZNep	RCC cell lines; Tumor xenograft in nude mice	Inhibit migration and invasion and up-regulate the expression of E-cadherin; Inhibit tumor growth and prolong survival	(Liu et al., 2016).
EZH2 siRNA	RCC cell lines	Prevent cell proliferation and invasion potential of 786-O cells	(Zhang et al., 2018).
EZH2 siRNA	RCC cell lines	Reduce the proliferation of RCC without inhibiting the tumor suppressor p27Kip1	(Sakurai et al., 2012)
shRNA EZH2, EPZ011989	RCC cell lines; Tumor xenograft in nude mice	Inhibit survival, invasion and growth of ccRCC cells with BAP1 mutation	(Sun et al., 2018)
EZH2 siRNA, 3-DZNeP	I/R induced AKI	Reduce acute kidney injury via targeting EZH2/p38 signaling pathway	(Liang et al., 2019)
EZH2 siRNA, 3-DZNeP	I/R or FA induced AKI	Reduce renal dysfunction and renal tubular cell death; Prevent renal tubular injury	(Zhou et al., 2018b)
EZH2 siRNA, 3-DZNeP	Cisplatin induced AKI	Suppress acute kidney injury via an E-cadherin-dependent mechanism	(Ni et al., 2019)
EZH2 siRNA, 3-DZNeP	I/R induced AKI	Alleviate I/R injury and block the activation of oxidative stress and pyroptosis	(Liu et al., 2020)
3-DZNeP, GSK126, EZH2 siRNA	Cultured NRK-49F cells; Mouse model of UUO	Attenuate fibrosis; Reduce the activation of renal interstitial fibroblasts	(Zhou et al., 2015)
3-DZNeP, EZH2 siRNA	Cultured TKPT cells; Mouse model of UUO	Attenuate renal fibrosis; Inhibit TGF-β1–induced EMT	(Zhou et al., 2018a)
3-DZNeP, EZH2 shRNA	Streptozotocin (STZ) -induced DN	Increase the glomerular TxnIP expression, induce podocyte injury, and increase oxidative stress and proteinuria	(Siddiqi et al., 2016)
EPZ-6438, EZH2 KO mice	Adriamycin nephrotoxicity; SNx	Sensitize mice to glomerular disease; Increase podocyte injury and dedifferentiation	(Majumder et al., 2018)
EZH2 siRNA	Rat MCs (RMC)s; Streptozotocin-induced rat model of type 1 diabetes and DN.	Destroy the low level, stable state of fibrosis and inflammatory genes in MCs. Inhibition and up-regulation of genes that cause glomerular MC and podocyte dysfunction	(Jia et al., 2019a)
EZH2 siRNA, 3-DZNeP, GSK126	HK-2cells; High-fat diet induced mice model of DN	Rescue SIRT1 expression and block ROS accumulation	(Zeng et al., 2018)
siEZH2, EPZ005687	Murine podocyte cell line; Streptozotocin-induced rat model of diabetes	Inhibit podocyte migration; reduce podocyte apoptosis	(Wan et al., 2017)
EZH2 siRNA, 3-DZNeP	NRK-49F and HK2 cells; Hyperuricemia-induced CKD	Decrease serum uric acid levels; Alleviate renal pathological damages	(Shi et al., 2019)
3-DZNeP	MRL/lpr mice	Reduce lupus associated kidney damage	(Rohraff et al., 2019)
3-DZNeP	T cells; mouse model of allogeneic bone marrow transplantation	Attenuate graft-versus-host disease (GVHD)	(He et al., 2012)
3-DZNeP	Rat model of renal transplantation	Ameliorate early acute renal allograft rejection	(Li et al., 2016)

## EZH2 and Renal Cell Carcinoma

Renal cell carcinoma (RCC), also known as renal adenocarcinoma. originates from renal tubular epithelial cells and accounts for over 90% of adult renal malignancies. The worldwide incidence RCC has increased significantly in recent years (Shah et al., 2009). At present, the cause and pathogenesis of RCC are not clear. Epidemiology speculates that the pathogenesis is related to genetics, smoking, obesity, hypertension, and antihypertensive treatment (Grossman et al., 1999). About 70% of renal cancers are associated with gene expression inactivation caused by VHL gene deletion and mutation. VHL gene encodes an E3 ubiquitin ligase complex protein, which can degrade hypoxia inducible factor (HIF). HIF is continuously activated in RCC cells, promoting the transcription of a series of downstream target genes (Mallikarjuna et al., 2018). Recently, it has been reported that epigenetic modification, in particular, EZH2 activation, participates in the occurrence and development of RCC, which provides a new direction for the treatment (Bannister and Kouzarides, 2011).

Studies have demonstrated that EZH2 can promote the development and metastasis of RCC. EZH2 is overexpressed in numerous tumor entities including renal tumor cells (Kim and Roberts, 2016; Sun et al., 2018). EZH2 overexpression leads to increases in H3K27me3, with repression of tumor-suppressor genes such as E-cadherin (Liu et al., 2016). *In vitro* and *in vivo* studies confirmed that the abnormal increase of EZH2 can inhibit the expression level of E-cadherin, induce the epithelial stromal transformation of renal cancer cells, and promote the occurrence, development and recurrence of renal cancer. Inhibition of EZH2 with 3-DZNep can reverse these pathological responses (Liu et al., 2016). EZH2 also has growth promoting activity in RCC (Wagener et al., 2010) and can enhance the proliferation and invasion of renal tubular epithelial cells (Zhang et al., 2018). Moreover, EZH2 promotes cell proliferation, migration and angiogenesis by inhibiting expression of tumor suppressor genes such as p27Kip1 and enhancing expression of proto-oncogenes (Sakurai et al., 2012). Thus, inhibition of EZH2 can reduce the survival and invasion of clear cell renal cell carcinoma (ccRCC) cells and the growth of ccRCC in xenografted mice (Sun et al., 2018). Finally, EZH2 in combination with the DNA methyltransferase DNMT can methylate the VHL promoter and inhibit its expression in RCC (Schlesinger et al., 2007).

## EZH2 and Acute Kidney Injury

Acute kidney injury is a common pathologic process with high mortality in hospitalized patients (Raimann et al., 2018). It can be caused by ischemia/reperfusion, septicemia, or nephrotoxins (i.e., radiocontrast agents, NSAIDs, etc.) (Zuk and Bonventre, 2016; Moledina et al., 2017; Wu et al., 2017). Acute injury to the kidney usually leads to death of renal tubular epithelial cells, activation of endothelial cells, infiltration of leukocytes, and ultimately renal dysfunction (Sato and Yanagita, 2018; Ronco et al., 2019). In mild injury, adaptive repair mechanisms can restore epithelial integrity, inhibit immune responses and reconstruct a healthy vascular system. On the contrary, severe or persistent damage can lead to inadequate repair (Sato and Yanagita, 2018; Ronco et al., 2019). Tubular cells may experience G2/M cell cycle arrest, senescence, apoptosis or necrosis, leading to release of pro-inflammatory factors (Guzzi et al., 2019). Emerging evidence has shown the role of EZH2-mediated histone modifications in AKI (Ho et al., 2017) (Bomsztyk and Denisenko, 2013).

The abnormal expression or activation of EZH2 is related to the pathogenesis of AKI Zhou et al., 2018b). Initially, it was found that EZH2 is involved in many cellular responses, such as apoptosis and inflammation (Wang Y. et al., 2018; Bamidele et al., 2019). The expression of EZH2 and H3K27me3 is up-regulated in ischemia-reperfusion and folic acid-induced AKI models (Zhou et al., 2018b). Inhibition of EZH2 with 3-DZNep can reduce renal dysfunction and tubular cell death (Zhou et al., 2018b). E-cadherin downregulation mediates disruption of cell-cell adhesion, activation of matrix metalloproteinases, and activation of ERK1/2, and it promotes activation of cell death receptor and regulation of mitochondrial damage. Inhibition of EZH2 can preserve expression of E-cadherin and tight junction protein ZO-1, inhibit expression of matrix metalloproteinase MMP-2 and MMP-9, and suppress phosphorylation of Raf-1 and ERK1/2 in renal tubular cells exposed to oxidative stress (Zhou et al., 2018b). This was confirmed by an *in vitro* study (Zhou et al., 2018b). In a murine model of cisplatin induced-AKI, inhibition of EZH2 expression by 3-DZNep could also reduce apoptosis of renal tubular cells and ameliorate acute renal injury by restoring expression of E-cadherin (Ni et al., 2019). Both *in vitro* and *in vivo*, cisplatin-induced renal tubular cell damage was accompanied by up-regulation of H3K27me3, while 3-DZNep treatment did not affect its expression (Ni et al., 2019). This suggests that 3-DZNeP-elicited renal protection in response to cisplatin exposure may not be through a H3K27me3-mediated mechanism. Recently, it was demonstrated that EZH2 can regulate renal injury by inducing oxidative stress as evidenced by the fact that EZH2 inhibition blocked the production of NOX4 dependent ROS through the ALK5/Smad2/3 signal pathway in an animal model of ischemia/reperfusion-induced AKI (Liu et al., 2020). In the same injury model, EZH2 inhibition also reduced renal dysfunction and tubular injury by regulating p38 signaling, apoptosis and inflammation (Liang et al., 2019). Therefore, EZH2 is an important mediator in the pathogenesis of AKI. Additional studies are needed to describe the mechanism of EZH2 in AKI in more detail.

## EZH2 and Renal Fibrosis

Renal fibrosis is a common pathological process in the progression of CKD to end-stage renal disease (ESRD). Renal interstitial fibrosis is considered an example of poor self-healing after injury (Iwano and Neilson, 1991; Inoue et al., 2015; Lovisa et al., 2015). It is mainly manifested by extracellular matrix (ECM) deposition, epithelial to mesenchymal transition (EMT), inflammatory response, and fibroblast activation and proliferation (Iwano and Neilson, 1991; Inoue et al., 2015; Lovisa et al., 2015). Although many studies have been conducted to elucidate its pathogenesis, there is still a lack of effective treatment for renal fibrosis (Boor et al., 2010; Nogueira et al., 2017).

Recent studies have demonstrated that EZH2 plays a critical role in the development of renal fibrosis. EZH2 expression levels are low in normal kidney tissue, but high in mice kidneys following injury and in human kidneys following disease (Zhou et al., 2015). Immunostaining showed that EZH2 was expressed both in tubular epithelial cells and renal interstitial fibroblasts (Zhou et al., 2015). Activation and proliferation of renal fibroblasts produces a large amount of ECM proteins, including fibronectin and collagen I. *In vitro*, pharmacological inhibition or siRNA mediated silencing of EZH2 reduced the activation of renal interstitial fibroblasts; *in vivo*, treatment with the EZH2 inhibitor 3-DZNep attenuated UUO-induced fibrosis in an animal model. The anti-fibrotic effect of EZH2 inhibition is related to the inhibition of expression of epidermal growth factor receptor (EGFR) and platelet-derived growth factor receptor (PDGFR) and deactivation of multiple intracellular signaling pathways, including TGFβ/Smad3, AKT and ERK1/2 (Zhou et al., 2015). EZH2 was also identified as a key regulator of epithelial-mesenchymal transition (EMT) in fibrotic kidneys (Zhou et al., 2018a). EZH2 inhibition elicits an anti-EMT effect related to preservation of E-cadherin expression, repression of transcription factors (i.e., Snail, twist), and deactivation of PTEN/Akt and β-catenin signaling pathways (Zhou et al., 2018a). Studies have also shown that EZH2 inhibition can effectively suppress the development of liver fibrosis (Zeybel et al., 2017), skin fibrosis (Tsou et al., 2019), atrial fibrosis (Song et al., 2019), pulmonary fibrosis (Xiao et al., 2016), and peritoneal fibrosis (Shi et al., 2020). These results suggest that EZH2 may serve as a promising therapeutic target for the treatment of fibrosis in many organ systems.

## EZH2 and Diabetic Nephropathy

Diabetic nephropathy is one of the most common chronic complications of diabetes (Alicic et al., 2017; Han et al., 2017; Feldman et al., 2019). The typical characteristics of DN are glomerular hypertrophy, proteinuria, progressive decrease of glomerular filtration rate, and renal fibrosis, which leads to loss of renal function. The pathological changes of diabetes are mainly composed of mesangial hyperplasia, thickening of basement membrane, occlusion of capillary lumen, disorder of podocyte structure, and decrease of podocyte number (Tung et al., 2018). However, the specific molecular mechanism of DN is not completely understood. Recent studies have shown that EZH2 activation is involved in the pathogenesis of DN. EZH2 can regulate oxidative stress in diabetic patients by inhibiting expression of Pax6, a transcription factor, and thus the expression of TxnIP, an endogenous antioxidant inhibitor (Siddiqi et al., 2016). The specific deletion of EZH2 in podocytes induced podocyte damage and dedifferentiation (Siddiqi et al., 2016) and sensitized mice to glomerular disease (Majumder et al., 2018). In human glomerular diseases such as focal segmental glomerulosclerosis and DN, H3K27me3 was decreased in podocytes. H3K27me3 and EZH2 are involved in inhibiting and maintaining the low-level and stable state of fibrosis and inflammation genes in mesangial cells, while H3K27me3 and EZH2 are inhibited by TGF-β, which increases the expression of genes that mediate glomerular mesangial dysfunction and DN, leading to renal dysfunction (Jia et al., 2019a). In contrast, the depletion or inhibition of EZH2 attenuated the increase of reactive oxygen species (ROS) in human renal tubular epithelial cells (HK-2) induced by high glucose (Zeng et al., 2018). Moreover, Wilm’s tumor 1 (WT1) can improve the β-catenin mediated damage of podocytes in DN by antagonizing EZH2, which is manifested in reducing the transformation of the stroma of DN podocytes, maintaining the structural integrity of DN podocytes, reducing apoptosis and oxidative stress of DN podocytes (Wan et al., 2017). These data illustrate that EZH2 activity is necessary for protection against podocyte damage in DN. This is opposite to what has been observed in the murine model of UUO-induced renal fibrosis (Zhou et al., 2015). Currently, it remains unclear about the underlying mechanism by which EZH2 plays distinct roles in different disease models and cells. Since multiple cell types are involved in the pathogenesis of DN, and EZH2 mediated gene regulation is cell-context specific, EZH2 may play the role of a double-edged sword in different cell types in DN (Brasacchio et al., 2009). Further investigation is required to elucidate the gene and signaling pathways regulated by EZH2 during DN development.

## EZH2 and Hyperuricemic Nephropathy

Hyperuricemia (HUA) is a purine metabolic disorder, in which the blood uric acid level is higher than the normal level due to the increase of uric acid production and/or the decrease of uric acid excretion. HUA is an independent risk factor for CKD progression (Mok et al., 2012) and has a direct correlation with renal damage (Lin et al., 2014). 10–20% of patients with primary hyperuricemia have evidence of AKI and chronic kidney disease, including chronic uric acid nephropathy, acute uric acid nephropathy and uric acid stones. Long-term HUA can cause renal damage through the urate crystal dependent pathway. Uric acid crystals are deposited in the distal collecting duct and the renal interstitium, causing chronic interstitial nephritis, which may lead to renal interstitial fibrosis (Liu et al., 2015; Yuan et al., 2017; Johnson et al., 2018). In addition, long-term HUA can cause renal damage through an independent pathway of urate crystal formation. In this regard, it has been reported that uric acid can cause renal endothelial dysfunction, renin-angiotensin system (RAS) activation, inflammation, oxidative stress (Sanchez-Lozada et al., 2008; Yu et al., 2010; Filiopoulos et al., 2012), whereas lowering serum uric acid alleviates renal damage or delays its progression (Obermayr et al., 2008; Zhou et al., 2012; Kim et al., 2014).

Although the molecular mechanism of renal damage caused by elevated uric acid levels remains obscure, uric acid has been shown to induce activation of TGF-β receptor and transcription of TGF-β1 target genes (Böttinger, 2007), leading to activation of downstream signaling pathways such as EGFR and ERK1/2 (Joo et al., 2008; Lee et al., 2010). Interestingly, blocking EZH2 by 3-DZNep inhibits TGF-β1-induced activation of renal interstitial fibroblasts *in vitro* and attenuates ECM protein deposition and α-smooth muscle actin expression in obstructed kidneys (Zhou et al., 2015). Inhibition of EZH2 by 3-DZNep also significantly reduces blood uric acid levels by reducing the activity of serum purine oxidase (XOD) and alleviates HUA-induced renal damage through various mechanisms, including inhibition of the TGF-β1/Smad3 and EGFR/ERK1/2 signaling pathways. Moreover, 3-DZNep was effective in downregulating the levels of various pro-inflammatory chemokines/cytokines and reducing the apoptosis of renal tubular cells (Shi et al., 2019). Therefore, EZH2 may be a potential therapeutic target for reducing renal damage and delaying the development of CKD caused by HUA.

## EZH2 and Lupus Nephritis

Systemic lupus erythematosus (SLE) is a chronic autoimmune inflammatory disease characterized by loss of immune tolerance to autoantigens, production of autoantibodies, formation of immune complexes and deposition in different parts of the body, causing inflammation and multiorgan damage (Marshall and Vierstra, 2018). LN is one of the most common and serious complications of SLE, affecting up to 60% of lupus patients by some estimates. LN is thus considered an important cause of chronic kidney disease (Koutsokeras and Healy, 2014; Zhu et al., 2016). The pathogenesis of SLE and LN is complex and exact mechanism(s) are largely unknown. It is generally believed that SLE is caused by genetic, endocrine, environmental factors (infection, ultraviolet radiation, etc.), and abnormal activation of the immune system. Epigenetic changes have also been reported to contribute to the pathogenesis of lupus (Ballestar et al., 2006). In particular, various abnormal patterns of DNA methylation of immune cell types isolated from lupus patients have been found to be related to clinical heterogeneity, interspecific disease variability, lupus onset and remission (Teruel and Sawalha, 2017).

Similar to DNA methylation, histone modifications can lead to abnormal gene expression and contribute to the pathogenesis of SLE (Hu et al., 2008). It has been reported that unregulated EZH2 activation in CD4+ T cells leads to T cell activation and non-Th1 immune responses, prior to transcription activity, and is related to lupus activity. In addition, levels of two microRNAs (miR-101 and miR-26a) targeting and regulating EZH2 in CD4+ T cells of lupus patients were negatively correlated with lupus disease activity (Coit et al., 2016). EZH2 can mark and control functions and survival of effector T cells through microRNAs and the Notch signaling pathway (Zhao et al., 2016). In lupus patients, overexpression of EZH2 leads to the methylation of JAM-A (junctional adhesion molecule A), which may increase the migration of T cells and lead to the invasive exosmosis of T cells. Blocking EZH2 by 3-DZNep and GSK126 can effectively inhibit the adhesion of lupus T cells to human microvascular endothelial cells. In addition, overexpression of EZH2 results in methylation changes of genes involved in gene transcription, ubiquitination and immune response, indicating that EZH2 is involved in various cellular and physiological processes crucial to the survival and function of T cells (Tsou et al., 2018). Inhibition of EZH2 by 3-DZNep significantly reduced the number of pathogenic double negative T cells and production of cytokines and chemokines in lupus prone MRL/lpr mice. Splenomegaly and lymphadenopathy in mice treated with 3-DZNep were significantly reduced. Most importantly, inhibition of EZH2 by 3-DZNep in MRL/lpr mice can reduce renal damage and increase survival rate of MRL/lpr mice. Mice with 3-DZNep treatment have relatively stable albumin:creatinine ratios, and attenuated glomerulonephritis and crescent formation. Glomerular necrosis in the prevention group of mice was significantly relieved as well. Therefore, 3-DZNep elicited inhibition of EZH2 can effectively prevent the progression of renal damage in lupus (Rohraff et al., 2019). Additional studies are necessary to examine whether other EZH2 inhibitors or gene modulators are also effective in ameliorating the pathogenesis of LN.

## EZH2 and Aging Kidney

Aging is an irreversible phenomenon characterized by gradual decline of cell function and gradual structural changes of many organ systems. Age-related changes in kidney function include anatomical and physiological changes (Zhou et al., 2008). The histological changes in renal aging mainly include: renal mass reduction, glomerulosclerosis, renal tubular atrophy, renal interstitial fibrosis and arterial intimal fibrosis (Silva, 2005). The partial loss of renal function can be manifested as decreases in renal vascular elasticity, renal blood flow and glomerular filtration rate. At present, mechanisms of renal aging are incompletely studied. Increasing evidence indicates that renal aging is related to epigenetic changes (Painter et al., 2008; Au et al., 2013; Kooman et al., 2014).

Epigenetic histone modification plays a role in aging (Sen et al., 2016), especially trimethylation of 27 lysine (H3K27me3) of histone H3, which is directly related to life span and aging in different models (Jin et al., 2011; Ma et al., 2018). Previous studies have shown that EZH2 expression is related to abnormal expression of genes in aging animal models (Shumaker et al., 2006; Bracken et al., 2007; Chen et al., 2009). For example, overexpression of EZH2 can prevent stem cell failure and aging (De Haan and Gerrits, 2007) while pharmacological inhibition of EZH2 can dysregulate tissue regeneration in aged mice (Nishiguchi et al., 2018). Methylation of CpG islands related to aging may overlap with the regulatory regions of cancer genes such as c1ql3. EZH2 interacts with these regulatory regions in mice, and the occupancy of EZH2 may decrease with age at c1ql3. EZH2 is part of the protein mechanism of forming the aging epigenome (Mozhui and Pandey, 2017). A recent study found that H3K27me3 regulated the expression of Klotho in the kidney of aging mice. A decrease of Klotho levels is an important mechanism of aging. The epigenetic down-regulation of Klotho gene expression is at least partly due to the histone 3 modification of the Klotho promoter. Aging plays a role by up-regulating H3K27me3 and down-regulating hyperphosphorylation of Klotho and mTOR in renal tubules. Inhibition of EZH2 with GSK343 or EED226 was able to reduce H3K27me3 recruitment to the Klotho promoter (Han and Sun, 2020). The expression level of EZH2 decreased in older mice (Han and Sun, 2020). At present, no study has confirmed the obvious correlation between the expression of EZH2 and H3K27me3 in the kidney of aging mice or adult tissues (Margueron et al., 2008). Studies have pointed out that renal aging can up-regulate the ECM laminin genes by down-regulating 5mC and H3K27me3 in the promoter region of the ECM laminin gene. Reduction of H3K27me3 levels by 3-DZNep can inhibit expression of the laminin gene in the ECM, but administration of a more specific EZH2 inhibitor, GSK-126, did not inhibit expression of the laminin gene in ECM (Denisenko et al., 2018). Therefore, the exact mechanism(s) by which EZH2 contributes to renal aging needs further investigation.

## EZH2 and Kidney Transplantation Rejection

Currently, renal transplantation is the most effective treatment for ESRD. However, acute rejection (AR) is a common adverse reaction after kidney transplantation, usually occurring weeks to months after transplantation. Rejection after transplantation is caused by the recipient’s immune system. Recognition of the graft as a foreign body stimulates secretion of various inflammatory factors to attack the graft. Eliminating the foreign body reaction and maintaining the stability of the internal environment would improve the long-term survival of the transplanted kidney. Transplantation rejection includes T-cell-mediated rejection and antibody mediated rejection (Haas et al., 2018). Acute T-cell mediated rejection is an inflammatory reaction, involving extensive T lymphocytes infiltration of the allograft and activation (Yang C. et al., 2015). T cell mediated AR of renal transplantation includes mononuclear interstitial infiltration and tubulitis with intima-intimal arteritis. Epigenetic modification is involved in T cell-mediated AR of renal transplantation (Cuddapah et al., 2010).

Enhancer of zeste homolog 2 plays an important role in maintaining T cell numbers and functions. It has been documented that EZH2 is essential for the expansion of T-effector cells (Yang X. P. et al., 2015) as well as differentiation and characteristic maintenance of regulatory T cells necessary for maintaining immune homeostasis (DuPage et al., 2015). EZH2 can also stimulate the expression of T cell multifunctional cytokines by activating the Notch pathway, and promoting T cell survival by Bcl-2 expression (Zhao et al., 2016). In addition, EZH2 can protect key T cell development regulators from DNA methylation so that they can be activated in a subsequent differentiation stage (Wang C. et al., 2018). Treatment with 3-DZNep, a EZH2 inhibitor can induce selective apoptosis of alloantigen-activated T cells and arrest persistent graft-versus-host disease (GVHD) in mice after allogeneic bone marrow transplantation (He et al., 2012). A recent study was the first to demonstrate the relationship between EZH2 and allograft rejection. EZH2 in T cells was increased after kidney transplantation in a rat model of kidney transplantation; inhibition of EZH2 by DZNep reduced AR, reduced injury and inflammatory infiltration of the transplanted kidney. The cellular mechanisms are related to the inhibition of activation and proliferation of alloreactive T cells, impairment of production of inflammatory factors, and increased apoptosis of alloreactive T cells in the transplanted kidney and peripheral blood) (Li et al., 2016). However, the specific mechanism(s) of EZH2 in AR of renal transplantation remains to be further studied.

## Conclusion and Perspectives

Enhancer of zeste homolog 2 is highly expressed in renal tumors and many kidney diseases. Abnormal expression or activation of EZH2 can lead to development and progression of renal tumors and several kidney diseases as indicated in this review. The molecular mechanisms of EZH2-mediated renal pathology are associated with renal tubular cell injury, podocyte dedifferentiation, renal interstitial fibroblast proliferation, production of multiple cytokines/chemokines and infiltration of inflammatory cells. Because EZH2 regulates expression of diverse genes and activation of multiple signaling pathways associated with pathogenesis of disease, its functional role may vary with cell types, tissues and disease models. In this context, EZH2 activation has been shown to contribute to renal tubular cell death, but protects against podocyte injury. Therefore, further studies are necessary to elucidate the detailed mechanistic actions of EZH2 in the pathogenesis and progression of kidney diseases.

Given that preclinical studies have demonstrated that EZH2 inhibitors attenuate some renal diseases in animal models, EZH2 inhibitors alone, or in combination with other drugs may provide beneficial effects to ameliorate or prevent kidney diseases. This is encouraged by recent approval of tazemetostat (EPZ-6438), one of EZH2 inhibitors, for treatment of adult patients with relapsed or refractory (R/R) follicular lymphoma by the FDA (Gulati et al., 2018). Among the 203 patients who were evaluated for efficacy, responses were seen in 24% patients who had received tazemetostat administered as a single agent in both tumor types and in EZH2 mutant and WT tumor (Gulati et al., 2018). In addition to tazemetostat, other EZH2 inhibitors such as GSK126 and CPT-1205 are being tested in treating lymphoma and several other solid tumor types (Eich et al., 2020). Currently, there are still no clinical trials of EZH2 inhibitors for the treatment of kidney disease of any underlying cause. Based on the evidence showing the efficacy of EZH2 inhibitors in animal models of kidney disease, clinical trials assessing the effect of EZH2 inhibition may hold out the promise of treatment for some forms of progressive kidney disease in humans. However, since genetic and pharmacological inhibition of EZH2 potentiates podocyte injury in murine models of glomerular disease (Majumder et al., 2018), EZH2 inhibitors may not be suitable for treatment of renal diseases associated with podocyte injury.

## Author Contributions

TL and CY drafted the article. SZ edited the manuscript. All the authors reviewed the manuscript and approved is for publication.

## Conflict of Interest

The authors declare that the research was conducted in the absence of any commercial or financial relationships that could be construed as a potential conflict of interest.
